# Effect of Long-Term Proton Pump Inhibitor Use on Glycemic Control in Patients with Type Two Diabetes Mellitus

**DOI:** 10.1155/2021/5578265

**Published:** 2021-07-29

**Authors:** Amy Trang, Jordan Bushman, Alexandra Halalau

**Affiliations:** ^1^Oakland University William Beaumont School of Medicine, Rochester Hills, MI, USA; ^2^Department of Internal Medicine, Rush University Medical Center, Chicago, IL, USA; ^3^Department of Internal Medicine, William Beaumont Hospital, Royal Oak, MI, USA

## Abstract

There have been conflicting results regarding the effect of proton pump inhibitors (PPIs) as an adjunctive therapy to oral antidiabetic medication (OAM) in those with type 2 diabetes (T2DM). PPIs increase gastrin levels, causing a rise in insulin. No studies have evaluated the duration of PPI therapy and its effect on glycemic control. Medical records across 8 hospitals between 2007 and 2016 were reviewed for 14,602 patients with T2DM (not on insulin therapy) taking PPIs. Values of HbA1c (baseline, follow-up, and the difference between the two) in those prescribed with PPIs and years of therapy were compared to HbA1c values of those who had no record of PPI use. Baseline and follow-up HbA1c for patients on PPIs were 6.8 and 7.0, respectively, compared to 7.1 and 7.2 in their untreated counterparts (*p* < 0.001 in both comparisons). For both groups, an increase in baseline HbA1c was seen with time. Those on PPI had an increase in HbA1c of 0.16 compared to 0.08 in those not prescribed PPI. Our results show no relationship between the length of PPI therapy and HbA1c reduction.

## 1. Introduction

Proton pump inhibitors (PPIs) are commonly prescribed in the United States, with a rise in patients using long-term therapy [1]. Guidelines recommend chronic treatment only be used for peptic strictures, erosive esophagitis, and Barrett's esophagus [2]. Some risks associated with chronic PPI therapy include dementia, chronic kidney disease (CKD), myocardial infarction (MI), fractures, and increased mortality [3]. The proposed mechanism by which PPIs improve HbA1c is by increasing serum gastrin which stimulates beta-cell neogenesis and causes an incretin-like effect, raising serum insulin [4]. However, there is conflicting evidence in the association between chronic PPI use and glycemic control. Two retrospective studies and one randomized control trial found an association between lower HbA1c levels while on PPI therapy [4–6]. In contrast, another randomized study and a meta-analysis were unable to show an effect on lowering HbA1c levels while on PPI therapy [3, 7]. We investigated whether there is a time-dependent relationship between PPI exposure and improvement in glycemic control in T2DM patients. We hypothesized that patients taking PPIs and oral antidiabetic medication (OAM) would have lower HbA1c values when compared to patients taking OAM alone. We also hypothesized that the longer the duration of PPI therapy, the lower the levels of HbA1c.

## 2. Design and Methods

This retrospective observational study includes patients from the Beaumont Health System (8 hospitals) between 2007 and 2016. The study was approved by the Beaumont Health Institutional Review Board, and informed consent was waived as the study was a retrospective data analysis. Inclusion criteria for patients were as follows: ICD 9 code diagnosis for T2DM, >18 years old, >one hemoglobin A1c measurement, and >one noninsulin OAM on their medication list. The treatment group includes patients with PPI therapy on their medication list for >one year. Exclusion criteria include documented use of insulin, corticosteroids, or H2 receptor blockers.

We collected data on demographics, duration of PPI therapy, CKD incidence, cardiac events, dementia, and mortality. HbA1c values were collected at baseline and follow-up to assess changes over time. Follow-up HbA1c values were all subsequently available values in the EMR during the study dates. ICD 9 codes were used to identify a diagnosis of CKD (any stage), dementia, and cardiac events (myocardial infarction, stroke, cardiac death). Mortality was noted by a deceased patient status in the electronic medical record. For statistical analysis, continuous variables were reported in mean (SD), *T*-test was used for continuous variables, chi-square was used for categorical variables, and regression analysis was used to adjust for the number of OAM.

## 3. Results

**Figure 1:**
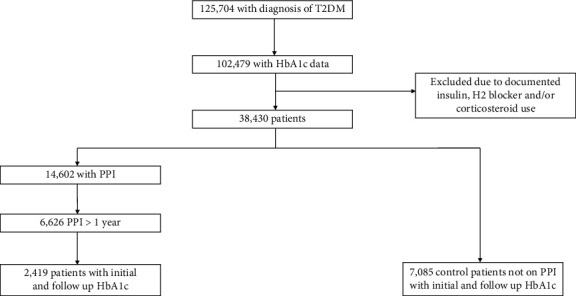
Flowsheet demonstrating the eligibility, inclusion, and exclusion criteria for patients in the study.

We included 2419 patients in the PPI+OAM group and 7085 patients in the OAM group (Figure 1). Baseline patient characteristics are shown in Table 1. Matched control patients were identified based on the distribution of HbA1c data which were similar between the groups (Table 1). In the treatment group, 37.5% were using PPIs for one year (Table 2). The mean duration of treatment of PPI therapy is discussed in Table 2, where most participants in either group were only on PPI therapy for one year.

**Figure 2:**
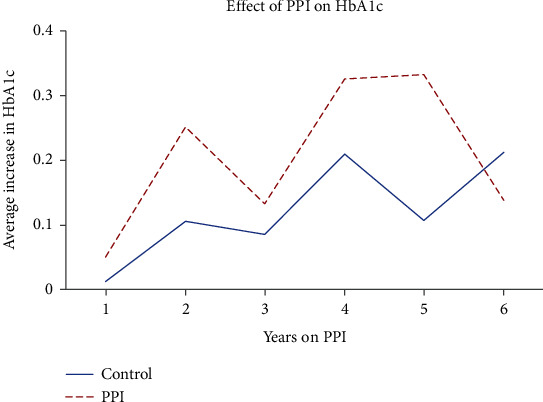
Trends of the changes in HbA1c values plotted across the time exposed to proton pump inhibitor treatment (in years).

Patients taking PPIs had significantly lower baseline HbA1c than those who were not (6.8 + 1.2 vs. 7.1 + 1.5, *p* ≤ 0.001). Follow-up HbA1c was also significantly lower in those on PPI therapy than their untreated counterparts (7.0 + 1.4 vs. 7.2 + 1.5, *p* ≤ 0.001). A regression analysis was used to adjust for the number of OAM while assessing the change in HbA1c with time. An increase from baseline HbA1c is seen with time and observed in both groups (0.16 ± 1.27 vs. 0.08 ± 1.48) (Figure 2).

Additionally, cardiac events (1.3% vs. 0.7%, *p* = 0.004), CKD (1.4% vs. 0.6%, *p* < 0.001), and mortality (6.2% vs. 4.8%, *p* = 0.005) were all significantly associated with PPI therapy use for at least one year. Dementia was not significantly associated with PPI use (0.5% vs. 0.3%, *p* = 0.223) (Table 3).

## 4. Discussion

Our study, encompassing a large population of T2DM patients, revealed lower mean baseline HbA1c and follow-up values in those on long-term PPI therapy. Our results show no relationship between the length of PPI therapy and HbA1c reduction. In fact, there was a general increase in HbA1c with time in both the PPI therapy group and control group. A possible explanation for low HbA1c values at year one could be that those who initiate PPI use are most adherent to daily dosing during their first year. Participant usage could have been intermittent (such as occasionally skipping doses) in subsequent years. The natural history of T2DM is a decline in beta-cell function, which may explain the general increase in HbA1c after the first year. The fact that HbA1c at year 6 (or the end of our follow-up in this study) was low is unclear. HbA1c at year 3 also declined from the prior year in both groups. It would be beneficial for future studies to assess PPI usage beyond 6 years as it would capture a more general pattern of HbA1c trends with more data points. Characteristics shown in Table 1 show baseline characteristics such as race, age, and BMI between the PPI and control group. This helps to show that the effect of PPI on HbA1c is more likely due to the intervention of PPI rather than preexisting differences between either groups.

Past studies evaluating PPI effects on glucose metabolism show conflicting results. A 12-week randomized control trial (RCT) demonstrated that pantoprazole significantly lowered HbA1c when compared to placebo [6]. A review of T2DM patients on PPI for >2 years and insulin therapy had significantly better glycemic control than those on insulin alone [4]. However, Takebayashi et al. were unable to show that a combination of PPIs and alogliptin was more effective than alogliptin alone on HbA1c levels during a 3-month period [7]. Similarly, a meta-analysis of 9 RCTs showed no significant effect of PPIs on glucose metabolism [3]. These studies varied in exposure time to PPIs and our study ultimately revealed no significant association with duration of PPI therapy and HbA1c values.

Interestingly, our treatment group had a greater incidence of cardiac events, CKD, and mortality. This may represent a direct effect of PPIs contributing to such disease states; however, the groups were not balanced based on the presence of comorbid conditions. A plausible reason for this finding may be that those on PPI therapy had more severe disease states and exacerbation of symptoms, requiring interventions. Another explanation for increased cardiac events may be due to PPIs competing with hepatic CYP450. This action prevents clopidogrel activation and increases the risk of clots and myocardial infarction [8]. CKD risk with PPI use has been quantified by Lazarus et al., demonstrating that PPI use was independently associated with a 20-50% greater risk of incident CKD [9]. Both cardiac events and incidence of CKD influence overall mortality; these patterns may explain our significant association with mortality in those on PPI therapy. Moreover, concerns of increased risk of bone fractures in the elderly with PPI use may also explain our mortality findings [10]. We had disproportionately more women in the PPI group; future studies investigating PPI effects on bone mineral density or fracture risk in T2DM patients are warranted to clarify this association.

There is conflicting evidence regarding whether PPI use is associated with dementia [11]. The proposed mechanism by which PPI use is associated with dementia is through the process of blocking V-type ATPases that degrade amyloid-B, a protein that predisposes to Alzheimer's disease [12]. As seen in Table 3, the results of our study show that dementia was not significantly associated with PPI use. The conflicting data surrounding this topic may be explained by the fact that those who begin PPI therapy could have more comorbidities than those who do not, especially in the elderly. Roughly, 65% of our participants in either group were younger than 70 years. In addition, it would have been helpful for our study to further cross match participants using a baseline comorbidity index such as the Charleston Comorbidity index. Further studies should consider using such indices to control for as much residual confounding as possible.

Our study has other limitations that would have helped with determining long-term influence of PPI on glycemic control. Future studies would benefit in analyzing basal C peptide values, type of PPI, and years from T2DM diagnosis. It would have also been useful to assess the number of OAD; however, given the multitude of OAD combinations, an inferential statistical analysis of this many groups in our study would not have been feasible. Due to such a large database, another limitation of our study is the inability to ascertain whether those in the control group used over-the-counter PPI on demand.

In conclusion, there is a mixed consensus regarding the effects of PPI therapy on glycemic control. Although our results show that baseline and follow-up HbA1c were lower in the PPI group, we did not find a relationship between the duration of PPI exposure and HbA1c reduction in T2DM patients. Risks of chronic PPI exposure when used as adjunctive therapy for T2DM patients should be carefully considered. Additional clinical trials are needed to further investigate whether the benefits of long-term PPI therapy outweigh their risks.

## Figures and Tables

**Table 1:** Baseline characteristics of the study participants.

	PPI (*n* = 2419)	Control (*n* = 7085)
Sex		
Male	940 (38.9%)	3597 (50.8%)
Female	1479 (61.1%)	3488 (49.2%)
Race		
White	1653 (68.3%)	4585 (64.7%)
Black	523 (21.6%)	1493 (21.1%)
Other	162 (6.7%)	646 (9.1%)
Missing	81 (3.3%)	371 (5.2%)
Age		
Under 40	82 (3.4%)	326 (4.6%)
40-50	263 (10.9%)	880 (12.4%)
50-60	504 (20.8%)	1736 (24.5%)
60-70	748 (30.9%)	2137 (30.2%)
70-80	518 (21.4%)	1342 (18.9%)
>80	304 (12.6%)	664 (9.4%)
BMI		
15-27	340 (14.1%)	1023 (14.4%)
27.1-30	341 (14.1%)	1013 (14.3%)
30.1-33	367 (15.2%)	1006 (14.2%)
33.1-38	382 (15.8%)	982 (15.9%)
38.1-82	407 (16.8%)	956 (13.5%)
Missing	582 (24.1%)	2105 (21.7%)

**Table 2:** Length (in years) of patients on PPI therapy and their matched controls. The control group was matched to those on PPI treatment with similar HbA1c data points. The percentages shown in the table demonstrate a similar distribution between both groups.

	PPI (*n* = 2419)	Control (*n* = 7085)
Years on PPI		
One	908 (37.5%)	3047 (43%)
Two	544 (22.5%)	1550 (21.9%)
Three	421 (17.4%)	1144 (16.1%)
Four	255 (10.5%)	614 (8.7%)
Five	180 (7.4%)	344 (4.9%)
Six	111 (4.6%)	386 (5.4%)

**Table 3:** Clinical outcomes of the study participants.

Outcome	PPI (*n* = 2419)	Control (*n* = 7085)	*p* value
Baseline HbA1c	6.82 ± 1.23	7.10 ± 1.48	<0.001
Follow up HbA1c	6.98 ± 1.36	7.17 ± 1.45	<0.001
Change in HbA1c	0.16 ± 1.27	0.08 ± 1.48	0.01
CKD	35 (1.4%)	45 (0.6%)	<0.001
Cardiac events	31 (1.3%)	47 (0.7%)	0.004
Dementia	12 (0.5%)	23 (0.3%)	0.223
Mortality	151 (6.2%)	339 (4.8%)	0.005

## Data Availability

The patient data used to support the findings of this study have not been made publicly available because of patient confidentiality.
